# Chest CT-Derived Muscle Analysis in COVID-19 Patients

**DOI:** 10.3390/tomography8010034

**Published:** 2022-02-08

**Authors:** Maurizio Antonarelli, Marco Fogante

**Affiliations:** 1 Azienda Sanitaria Locale Lanciano, 66100 Vasto, Italy; 2 Department of Radiology, Azienda Ospedaliero-Universitaria “Ospedali Riuniti”, 60126 Ancona, Italy; marco.fogante89@gmail.com

**Keywords:** COVID-19, sarcopenia, CT, CT-derived muscle analysis, intensive care unit, pneumonia

## Abstract

*Background:* sarcopenia is a predictor of unfavorable outcomes, but its prognostic impact on patients with COVID-19 is not well known. To evaluate the association between the chest computed tomography (CT) derived muscle analysis of sarcopenia and clinical-radiological outcomes in coronavirus disease 2019 (COVID-19). *Methods:* in this retrospective study were revised the medical records of patients admitted to the intensive care unit (ICU) and intubated for COVID-19. All patients had undergone chest CT scan prior to intubation, and the cross-sectional areas of the pectoralis muscles (PMA, cm^2^) and density (PMD, HU) were measured at the level of the fourth thoracic vertebral. The relationship between PMA and PMD and CT severity pneumonia, length of ICU, extubation failure/success, and mortality were investigated. *Results:* a total of 112 patients were included (82 M; mean age 60.5 ± 11.4 years). Patients with successful extubation had higher PMA compared to patients with failure extubation, 42.1 ± 7.9 vs. 37.8 ± 6.4 cm^2^ (*p* = 0.0056) and patients with shorter ICU had higher PMA and PMD compared to those with longer, respectively, 41.6 ± 8.7 vs. 37.2 ± 6.7 cm^2^ (*p* = 0.0034) and 30.2 ± 6.2 vs. 26.1 ± 4.9 HU (*p* = 0.0002). No statistical difference in PMA and PMD resulted in CT severity pneumonia and mortality. *Conclusion:* sarcopenia in COVID-19 patients, evaluated by CT-derived muscle analysis, could be associated with longer ICU stay and failure extubation.

## 1. Introduction

Coronavirus disease 2019 (COVID-19) caused by severe acute respiratory syndrome coronavirus-2 (SARS-CoV-2) is clinically characterized by different symptoms, such as fever, cough, dyspnea, fatigue, and myalgia, and can evolve into severe pneumonia, acute respiratory distress syndrome, and death [1,2].

Sarcopenia is an age-related clinical syndrome characterized by the progressive loss of skeletal muscle mass and strength [3]. Sarcopenia begins to develop around middle age, often due to an unbalanced or poor diet and low levels of physical activity, as well as chronic pathologies such as obesity and diabetes mellitus. Early sarcopenia is characterized by a decrease in the size of muscle. Over time, a reduction in muscle tissue quality also occurs. This is characterized by the replacement of muscle fibers with fat, an increase in fibrosis, changes in muscle metabolism, oxidative stress, and degeneration of the neuromuscular junction. This ultimately leads to progressive loss of muscle function and to frailty. This irreversible process causes muscle tissue deficits and compromises the quality and functionality of the striated muscle, which are replaced by adipose tissue and fibrosis [4]. Sarcopenia is an unfavorable independent predictor prognostic factor in numerous diseases, resulting in a worse prognosis under conditions such as major surgery and oncological and cardiovascular disease [5]. Moreover, sarcopenia is related to an increased risk of infections, a prolonged hospital stay, and an overall reduction in overall survival [6]. Sarcopenia also affects those muscles with respiratory functions, especially ventilation. In the elderly, the aging of the rib cage and the reduced elasticity of the lung tissue increase the work of the expiratory and inspiratory muscles, and sarcopenia can impair the ability to produce adequate respiratory volumes and to perform high-force airway removal maneuvers, such as extubation after mechanical ventilation. It has long been thought that the age-related loss of weight, along with a loss of muscle mass, was largely responsible for muscle weakness in older people. However, studies in patients with SO reveal that changes in muscle composition are also important. ‘Marbling’, or fat infiltration into muscle, lowers muscle quality and work performance. Studies to understand the pathogenesis of SO observed certain patterns of age-related changes in body muscle and fat composition [7].

During the current COVID-19, the elderly populations, which have increased considerably in recent decades, have revealed a wide vulnerability with an increasing hospitalization and mortality. Moreover, given that social isolation is adopted as the most protective measure against COVID-19, the level of physical activity and the intake of adequate diet have considerably declined, especially among older adults-denoting an increased possibility for developing sarcopenia [8].

Recent guidelines recommend the use of muscle strength tests using simple measures such as grip strength to identify individuals with muscle weakness and who need to undergo muscle mass testing [9,10]. Muscle mass is measured by various techniques such as dual-energy x-ray absorptiometry, ultrasound, magnetic resonance imaging, and computed tomography (CT). In particular, CT allows to accurately differentiate the fatty from muscle tissue using the specific attenuation for each type of tissue, and it provides very detailed anatomical information. Some studies have shown that the area of the pectoralis muscles evaluated on cross-sectional computed tomography (CT) images is associated with sarcopenia, and this information on muscle status could be easily retrieved by segmentation with a CT-derived muscle analysis on chest-CT, which has been extensively used for COVID-19 patients [9,10].

To date, the impact of sarcopenia in COVID-19 patients has only been preliminarily assessed, and few studies evaluated the association between CT-muscle analysis of sarcopenia and COVID-19 patients. Therefore, the aim of this work is to investigate the association between the CT-derived muscle analysis of sarcopenia and clinical-radiological outcomes in COVID-19 pneumonia.

## 2. Materials and Methods

This study was approved by the Hospital Institutional Review Board of Azienda Sanitaria Locale, and written informed consent was waived.

### 2.1. Study Population

In this retrospective study were revised the medical records of 127 patients admitted to the intensive care unit (ICU) and intubated for COVID-19 pneumonia between January to September 2021. The inclusion criteria were the presence of an unenhanced chest CT within seven days before the intubation. The exclusion criteria were patients younger than 18 years old (*n* = 4), major motion artifacts on CT (*n* = 6), and patients whose pectoral muscles were not included in the field of view on chest CT images (*n* = 5).

According to the exclusion and inclusion criteria, the final population was composed of 112 patients.

### 2.2. Clinical Evaluation

The presence of symptoms before the ICU admission, such as fever, cough, dyspnea, and myalgia or fatigue, were collected.

The presence of comorbidities before the ICU admission, such as hypertension, congestive heart failure, diabetes, chronic lung disease, obesity, and tumor history, was noted. Besides, smoking status was recorded in all patients.

All patients were followed through 31 September 2021, and the length of ICU stay, the success or failure extubation, and death were recorded.

### 2.3. Chest CT Image Acquisition

Chest CT images were obtained using a multi-detector CT scanner (GE Optima 660, GE Healthcare) at deep inspiration in the supine position. The CT parameters were: 128 × 0.35 mm slice collimation, 0.4 s rotation time, 1.5 mm slice thickness and 1 mm slice reconstructions, 250–300 mm field of view, 90−120 kV tube voltage, 50–90 mAs effective tube current-time product, and a 512 × 512 matrix.

### 2.4. Chest CT Derived Muscle Analysis

The images were evaluated in consensus by two radiologists with five years of experience. All CT images were anonymized prior to evaluation. On a single axial chest CT image at the level of the fourth thoracic vertebral, the pectoralis muscle area (PMA, cm^2^), and the pectoral muscular density (PMD, HU) were measured by a trained radiologist using a free DICOM viewer (Horos software Version 3.3.3, Norwich, England). Both of the pectoralis major and minor muscles were manually shaded using a predefined attenuation range of −50 and 90 HU because it is the range of the attenuation value for the muscular and intermuscular adipose tissues. Bilateral muscle areas were automatically measured between those CT attenuation values. PMA value was obtained by summing bilateral pectoralis major and minor muscle areas. PMD value was obtained automatically by software analysis.

### 2.5. Chest CT Lung Evaluation

In the chest CT image evaluation, lung window settings (with a window center of −600 HU and a window width of 1500 HU) were used to evaluate the pneumonia severity score (PSS) in each patient. The involvement severity was evaluated separately for each lobe, and the scores found for each lobe were summed for obtaining the PSS. The percentage of each lobe involvement was calculated as follows: no involvement (0%: 0 points), minimal involvement (1–25%: 1 point), mild involvement (26–50%: 2 points), moderate involvement (51–75%: 3 points), severe involvement (76–100%: 4 points). Two radiologists with five years of experience evaluated, independently, the images. In the presence of disagreement between the two observers for the PSS score, these CT images were reevaluated by a third observer with 22 years of experience in thoracic imaging, and an agreement was reached.

### 2.6. Statistical Analysis

The statistical analysis was performed using MedCalc (MedCalc Software Ltd., Ostend, Belgium). Categorical data were presented as a number and proportion (%), continuous data as mean with standard deviation (SD).

The relationship between PMA and PMD and PSS, length of ICU, extubation failure/success, and mortality were investigated. Categorical and continuous data were compared using a chi-squared and the Mann-Whitney U test.

To estimate the relative effect of variables by calculating unadjusted odds ratios (ORs) for categorical outcomes, logistic regression analysis was used.

Statistically, a *p*-value of <0.05 was considered significant.

## 3. Results

### 3.1. Study Population

One hundred and twelve patients with COVID-19 were enrolled in the study according to the exclusion and inclusion criteria.

There were 82 males (73.2%) and 30 females (26.8%) with a mean age of 60.5 ± 11.4 years. Before ICU admission and intubation, 39 (45.3%) patients had one comorbidity, 21 (24.4%) had two comorbidities, 16 (18.6%) had three comorbidities, and 10 (11.6%) patients more than three. The most common comorbidities were hypertension in 33 patients (29.5%), obesity in 32 patients (28.6%), and diabetes in 28 patients (25.0%).

The prevalent symptoms before ICU admission and intubation were fever in 77 patients (68.8%) and cough in 61 patients (54.5%). Twenty-five (22.3%) patients were smokers. Table 1 summarizes the clinical features of the study population.

### 3.2. CT-Derived Muscle Analysis and Clinical-Radiological Outcome Comparison

The median PSS was 7, and patients with a PSS < 7 were accepted as low PSS; instead, patients with a PSS > 7 were considered as high PSS. The median ICU stay was 19 days, and patients with an ICU stay < 19 were accepted as short ICU stay; instead, patients with an ICU stay > 19 were considered as long ICU stay.

Patients with successful extubation had higher PMA compared to patients with failure extubation, 42.1 ± 7.9 cm^2^ vs. 37.8 ± 6.4 cm^2^ (*p* = 0.0056).

Differently, no statistically significant differences were noted in PMD between patients with successful and failed extubation, respectively, 28.2 ± 5.7 HU vs. 27.1 ± 5.3 HU (*p* = 0.3295).

Patients with shorter ICU had higher PMA compared to those with longer, respectively, 41.6 ± 8.7 cm^2^ vs. 37.2 ± 6.7 cm^2^ (*p* = 0.0034). In the same way, patients with shorter ICU had higher PMD compared to those with longer, respectively, 30.2 ± 6.2 HU vs. 26.1 ± 4.9 HU (*p* = 0.0002).

No statistical difference in PMA and PMD resulted in CT severity pneumonia and mortality. Indeed, patients with high and low CTT severity pneumonia had PMA, respectively of 40.4 ± 9.8 cm^2^ vs. 38.9 ± 7.8 cm^2^ (*p* = 0.3721) and had PMD, respectively of 29.2 ± 5.9 HU vs. 27.8 ± 5.2 HU (*p* = 0.1856).

No statistical difference in PMA and PMD resulted in mortality.

After the adjustment with the prevalent comorbidities, such as hypertension, obesity, and diabetes, decreasing of PMA was found to be an independent predictor of prolonged ICU stay (OR: 1.69, 95% CI 1.23−2.19, *p* < 0.0292) and successful extubation (OR: 1.22, 95% CI 1.11−1.35, *p* < 0.0109). The results are summarized in Table 2.

**Figure 1:**
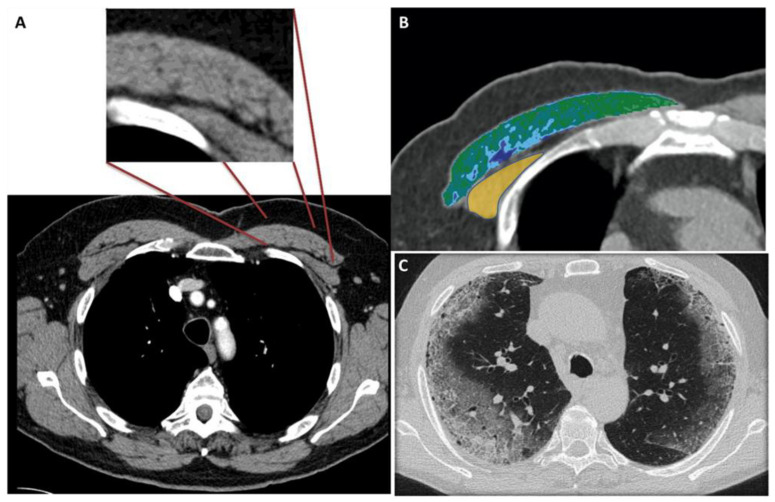
(**A**–**C**) 56-year-old male with COVID-19.

**Figure 2:**
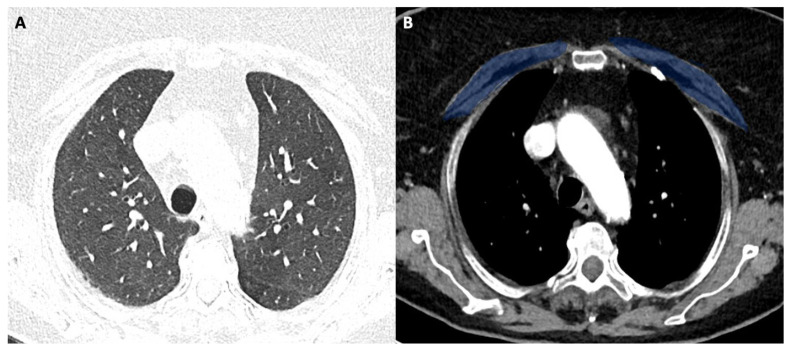
(**A**,**B**) 58-year-old female with COVID-19.

**Figure 3:**
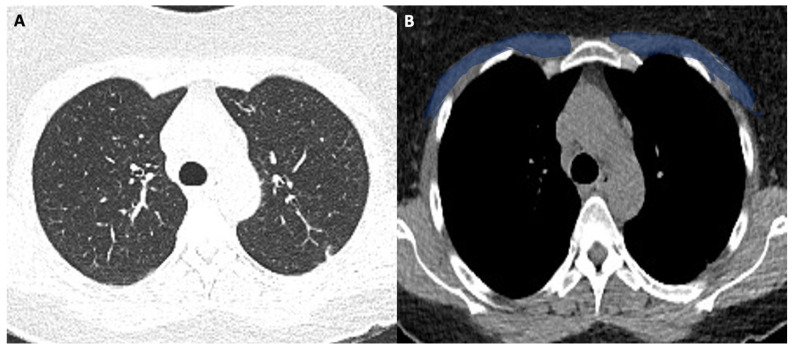
(**A**,**B**). 63-year-old male with COVID-19.

Figure 1 shows the modality of PMD and PMA evaluation. Figure 2 and Figure 3 show the examples of two patients enrolled in the study.

Figure 1A shows the CT-slice selection and the pectoral analysis. Figure 1B shows the pectoral muscle area (34.2 cm^2^) and pectoral muscle density (30.2 HU) analysis of the pectoralis major (green-blue area) and minor (yellow area). Figure 1C shows the corresponding lung disease (pneumonia severity index score: 7). The patient had a short ICU stay, and he had successful extubation.

Figure 2A shows the CT image with lung window (pneumonia severity index score: 5). Figure 2B shows the corresponding level in the mediastinal window with high pectoral muscle area (blue area). The patient had a long ICU stay, and he had successful extubation.

Figure 3A shows the CT image with lung window (pneumonia severity index score: 8). Figure 3B shows the corresponding level in the mediastinal window with a low pectoral muscle area (blue area). The patient had a long ICU stay, and he had extubation failure.

## 4. Discussion

Sarcopenia is a clinical syndrome, mainly due to aging, characterized by a reduction in muscle mass and density associated with a deficit of strength and endurance [11]. It begins around middle age, it derives from an unbalanced or poor diet and low levels of physical activity, and it is associated with chronic pathologies such as obesity and diabetes mellitus. CT imaging is an emerging novel modality to provide an estimate of an individual’s degree of sarcopenia by measuring the cross-sectional area of skeletal muscle on a single thoracic slice [12]. The present study included intubated patients and showed that patients with successful extubation had higher PMA compared to those with failure.

A possible explanation is that sarcopenia impairs the ability to produce adequate respiratory volumes after mechanical ventilation. In fact, sarcopenia of the accessory respiratory muscles and also of the diaphragm after a prolonged intubation time may cause a slower recovery of spontaneous breathing [13].

Patients with shorter ICU had higher PMA (*p* = 0.0034) and PMD (*p* = 0.0002) compared to those with longer, probably because patients with a reduced muscle mass need more time to overcome the cytokine “storm” caused by the virus [14]. No statistical difference in PMA and PMD resulted in inpatient mortality. This result could be explained because COVID-19 can cause serious complications and death even in young individuals. Moreover, it is important to point out that patients with obesity and diabetes often suffer complications and death, and these individuals are usually classified as no-sarcopenic. Finally, no statistical difference in PMA and PMD were in CT severity pneumonia, probably because the CT severity strictly depends on the time in which it is performed with possible sudden clinical and radiological worsening. Despite its clinical importance, sarcopenia remains under-recognized and poorly used in clinical routines due to the lack of available diagnostic tests and uniform diagnostic criteria; indeed, until now, there are no cut-off values in CT to distinguish sarcopenic and no-sarcopenic patients [15]. However, several scientific papers have highlighted the role of CT-derived muscle analysis in estimating the degree of sarcopenia and the correlation with clinical outcomes in various pathological conditions and in COVID-19 [16]. Rozenberg et al. [17] found that sarcopenia relates to hospitalization time and mortality in lung transplant patients. Kumar et al. [18] showed that the degree of sarcopenia correlates negatively with overall mortality in patients with advanced ovarian cancer. Kinsey et al. [19] concluded that a low CT area of the pectoral muscles is associated with reduced survival in patients with non-small cell lung cancer. Moon SW et al. [20] showed that the reduction in thoracic muscle mass correlates to mortality in patients with idiopathic pulmonary fibrosis. In COVID-19 patients, Damanti et al. [21] showed that muscle mass is associated with successful extubation, shorter ICU stay, and lower muscle density is associated with a longer length of hospitalization. These conclusions confirm our results. Schiaffino et al. [22] concluded that in hospitalized patients with COVID-19, lower muscle mass on CT images was independently associated with intensive care unit admission and in-hospital mortality. Differently to us, they concluded that lower muscle mass on CT images was independently associated with in-hospital mortality. Kim et al. [23] demonstrated that patients with sarcopenia showed a longer time to discharge and a higher incidence of death than those without sarcopenia. Finally, similar to us, Ufuk et al. [24] showed that PMA value is a predictor of prolonged hospital stay. To date, CT represents the simplest and most promising imaging modality in the assessment of sarcopenia, although it takes time in muscle segmentation. Some problems also arise due to the absence of currently validated thresholds for semi-automatic segmentation. However, radiologists can be very important for the evaluation of sarcopenia, especially because they can evaluate the impact of the same in different pathologies and ultimately in COVID-19.

Moreover, several studies suggest that obesity is a strong risk factor for adverse outcomes in patients hospitalized with COVID-19. Low skeletal muscle mass and sarcopenia are known risk factors for cardiometabolic disease, longer hospital stay, and increased mortality; and fat between muscle cells, intermuscular adipose tissue has been identified as an independent risk factor of impaired glucose tolerance, impaired lipid profile, chronic inflammation, and lower muscle strength and quality. All these deleterious mechanisms must be swiftly put to a check by a multidisciplinary approach, including nutritional support, early physical as well cardio-pulmonary rehabilitation, and psychological support and cognitive training.

This study had several limitations. First, the study has been retrospectively performed in a single hospital. Second, the sample size was relatively small. Third, the study population was treated within a single medical center, and all patients were from a single geographic region.

In conclusion, the SARS-CoV-2 pandemic has posed a challenge to health systems around the world. The ICU is crucial in patient management to improve outcomes and to reduce mortality, and imaging plays a fundamental role in lung disease diagnosis and evaluation. Low PMA could be predictive for failure extubation, and long ICU stay instead low PMD could be predictive only for the latter.

However, our study showed that the estimation of the degree of sarcopenia with a CT-derived analysis could represent a prognostic element in COVID-19 patients, and chest-CT could have a role not only in the evaluation of the lung but also in the assessment of sarcopenia, that radiologist could carry out and report.

## Figures and Tables

**Table 1:** Clinical features of the study population.

Features		All Patients (*n* = 112)
**Age (years)**		60.5 ± 11.4
**Sex**		
	Male	82 (73.2%)
	Female	25 (22.8%)
**Symptoms**		
	Fever	77 (68.8%)
	Cough	61 (54.5%)
	Dyspnea	35 (39.2%)
	Myalgia or fatigue	46 (41.1%)
**Comorbidities**		
	Hypertension	33 (29.5%)
	Congestive heart failure	27 (24.1%)
	Diabetes	28 (25.0%)
	Chronic lung disease	25 (22.3%)
	Obesity	32 (28.6%)
	Smoking History	25 (22.3%)
	Tumor History	20 (17.9%)

**Table 2:** CT-derived muscle analysis comparison in clinical and radiological features.

Features	Number of Patients	PMA (cm^2^)	PMD (HU)
**PSS**			
Low (<7)	56 (50%)	40.4 ± 9.8	29.2 ± 5.9
High (>7)	56 (50%)	38.9 ± 7.8	27.8 ± 5.2
*p*-value		0.3721	0.1856
**Length of ICU (days)**			
Short (<19)	56 (50%)	41.6 ± 8.7	30.2 ± 6.2
Long (>19)	56 (50%)	37.2 ± 6.7	26.1 ± 4.9
*p*-value		0.0034	0.0002
**Extubation**			
Successful	77 (68.8%)	42.1 ± 7.9	28.2 ± 5.7
Failure	35 (31.3%)	37.8 ± 6.4	27.1 ± 5.3
*p*-value		0.0056	0.3295
**Death**			
No	83 (74.1%)	39.9 ± 8.1	28.4 ± 5.6
Yes	29 (25.9%)	39.1 ± 7.8	26.9 ± 5.2
*p*-value		0.6449	0.2089

Abbreviations—PSS: pneumonia severity index; ICU: intensive care unit; PMA: pectoral muscle area; PMD: pectoral muscle density; HU: Hounsfield Unit.

## Data Availability

Not applicable.
